# Etiology, incidence, and outcomes of patient–ventilator asynchrony in critically-ill patients undergoing invasive mechanical ventilation

**DOI:** 10.1038/s41598-021-90013-z

**Published:** 2021-06-11

**Authors:** Yongfang Zhou, Steven R. Holets, Man Li, Gustavo A. Cortes-Puentes, Todd J. Meyer, Andrew C. Hanson, Phillip J. Schulte, Richard A. Oeckler

**Affiliations:** 1 Division of Pulmonary and Critical Care Medicine, Mayo Clinic, 200 1st St SW, Rochester, MN 55902 USA; 2 Department of Critical Care Medicine, West China Hospital, Chengdu, China; 3 Department of Respiratory Therapy, Mayo Clinic, Rochester, MN USA; 4 Department of Information Technology, Mayo Clinic, Rochester, MN USA; 5 Department of Health Science Research, Mayo Clinic, Rochester, MN USA

**Keywords:** Respiratory distress syndrome, Respiratory tract diseases, Respiration, Outcomes research, Respiratory signs and symptoms, Risk factors

## Abstract

Patient–ventilator asynchrony (PVA) is commonly encountered during mechanical ventilation of critically ill patients. Estimates of PVA incidence vary widely. Type, risk factors, and consequences of PVA remain unclear. We aimed to measure the incidence and identify types of PVA, characterize risk factors for development, and explore the relationship between PVA and outcome among critically ill, mechanically ventilated adult patients admitted to medical, surgical, and medical-surgical intensive care units in a large academic institution staffed with varying provider training background. A single center, retrospective cohort study of all adult critically ill patients undergoing invasive mechanical ventilation for ≥ 12 h. A total of 676 patients who underwent 696 episodes of mechanical ventilation were included. Overall PVA occurred in 170 (24%) episodes. Double triggering 92(13%) was most common, followed by flow starvation 73(10%). A history of smoking, and pneumonia, sepsis, or ARDS were risk factors for overall PVA and double triggering (all *P* < 0.05). Compared with volume targeted ventilation, pressure targeted ventilation decreased the occurrence of events (all *P* < 0.01). During volume controlled synchronized intermittent mandatory ventilation and pressure targeted ventilation, ventilator settings were associated with the incidence of overall PVA. The number of overall PVA, as well as double triggering and flow starvation specifically, were associated with worse outcomes and fewer hospital-free days (all *P* < 0.01). Double triggering and flow starvation are the most common PVA among critically ill, mechanically ventilated patients. Overall incidence as well as double triggering and flow starvation PVA specifically, portend worse outcome.

## Introduction

Invasive mechanical ventilation is essential for the treatment of critically ill patients with respiratory failure, yet a double-edged sword with potential to harm if ventilator settings do not meet patient demand. Suboptimal or mismatched ventilation selection may lead to patient ventilator asynchrony (PVA). PVA is common and may occur at any point during the course of mechanical ventilation. Its reported incidence varies widely, ranging from 10 to 85%^1–5^.

The factors that affect the occurrence of PVA can be related to the patient, the ventilator, or both. Patient factors include severity of illness, underlying diagnosis, indication for mechanical ventilation, and patient response to medical treatments^3,6^. Several studies^3,7–10^ have shown that patients with chronic obstructive pulmonary disease (COPD), in the presence of intrinsic PEEP, experience ineffective triggering or wasted efforts, delayed triggering, or prolonged cycling asynchronies. Another common PVA, double triggering, more frequently occurs in acute respiratory distress syndrome (ARDS) patients ventilated with low tidal volume^11^. The choice of ventilator mode and settings may also play a role^3^.

PVA may result in adverse consequences including increased respiratory workload, patient discomfort, deterioration in gas exchange, diaphragmatic injury, and/or patient self-inflicted lung injury. PVA is associated with worse clinical outcomes objectively identified by increased duration of mechanical ventilation, length of intensive care unit (ICU) and hospital stay, and mortality^12–18^. To date studies of PVA have mainly examined specific patient populations and/or ventilator modes and been limited by the timing and length of observations. The risk factors for, and clinical consequences of, PVA remain unclear. Though some may be benign, growing evidence suggests that certain PVAs may be more deleterious in the ventilatory management of patients with acute respiratory failure^12,16,18,19^.

Advanced methods of detecting PVA include esophageal pressure monitoring, diaphragm electrical activity, and software algorithms to continuously and automatically detect PVA. These techniques can provide robust information and help clinician detection of PVA but are quite complex to employ, requiring dedicated equipment and specialized expertise. As such these tools are not routinely used in clinical practice^6,20–24^. Furthermore, automated PVA detection has not been validated in large, heterogeneous populations and remains restricted mostly to research protocols^6,21,24,25^.

In contrast, ventilator waveforms require no additional equipment and are readily available for real-time, examination and interpretation by experienced clinicians^26,27^. In current clinical practice, the most frequent and practical approach to detect PVA remains the evaluation of airway pressure, flow, and volume tracings on the ventilator display. Utilizing our local standardized mechanical ventilation practice and incorporating an established, routine PVA management by bedside respiratory therapists, we aimed to systematically investigate the prevalence of and identify risk factors for PVA, as well as define associations between PVA and clinical outcome in critically ill mechanically ventilated patients.

## Methods

### Study design and patients

This was a single-center, retrospective cohort study. From December 2018, through May 2019, adult patients admitted to the medical and surgical ICUs, Mayo Clinic, Rochester, MN, and requiring invasive mechanical ventilation for at least 12 h, were included. Patients less than 18 years of age, with no evidence of spontaneous breathing, in a moribund state or palliative care were excluded. The study was reviewed and approved by the Mayo Clinic Institutional Review Board. All research was performed in accordance with relevant guidelines/regulations, and informed consent was obtained from all participants and/or their legal guardians.

### Definition of PVA and counting PVA events

In October 2018, before study commencement, all Respiratory Therapists received PVA identification training consisting of analysis of the ventilator’s pressure, flow, and volume waveforms. Following the training, RTs were required to pass a PVA identification competency exam with a minimum score of 85% detailed in Supplementary Information S1. Our institution has a standardized mechanical ventilation guideline which includes respiratory therapists performing a 10-min assessment of all mechanically ventilated spontaneously breathing patients to identify the occurrence of PVA. Assessments were conducted every 4 h during routine ventilator checks or sooner if the RT felt an assessment was warranted. The presence and type of PVA were documented in the electronic medical record (EMR).

Definitions of various types of PVA were derived from descriptions in the literature as follows^12,16,27–30^. Double-triggering: Sustained patient inspiratory effort beyond the ventilator inspiratory time resulting in the triggering and delivery of all or part of a second ventilator breath. Flow starvation: Ventilator inspiratory flow rate does not meet patients’ inspiratory demand. In volume controlled ventilation flow starvation is represented by a concave deflection (scalping out) of the pressure–time tracing during early inspiration with a square flow profile or a concave deflection in late inspiration with a decelerating flow profile. In pressure controlled ventilation flow starvation is due to a slow pressurization rate (rise time) and represented by a distortion (rounding off or shoulder) of early part of the pressure waveform. Ineffective triggering (wasted effort): Patient inspiratory effort prior to complete exhalation that does not initiate a ventilator breath. Represented by a temporary decrease in expiratory flow prior to the next delivered breath. Cycle mismatch including premature cycling and prolonged cycling. Premature cycling: Inspiratory effort continues beyond ventilator inspiratory time represented by a decrease in expiratory flow and airway pressure immediately after the onset of expiration; Prolonged cycling: Ventilator inspiratory time is longer than the patient’s effort, represented as a sharp spike at the end of inspiration. Delayed Triggering: A delayed response of the ventilator output to patient inspiratory effort. Represented by a marked decrease in pressure before the ventilator output begins. Examples of asynchronies are available in Supplementary Information S1. Overall PVA included any type of PVA listed as above.

### Data collection

Demographics, mechanical ventilation data, and clinical outcomes were extracted from EMR, listed as the following: (1) Demographics, chronic comorbidities; (2) Reasons for initial mechanical ventilation; (3) PVA assessment: the prevalence of any type of PVA; (4) Ventilator mode and ventilator settings at the time of PVA assessment, and the classification of ventilator modes listed in Supplementary Information S2; (5) Clinical outcomes: mechanical ventilation duration, ventilator-free days at day 28, length of ICU and hospital stay, ICU and hospital mortality. Ventilator free days at day 28 were defined as the number of ventilator-free days and alive through 28 days after mechanical ventilation initiation^31^. All data were collected during the first episode of mechanical ventilation during the first ICU stay, whether or not multiple episodes occurred during the same hospital stay.

### Statistical analysis

Incidence of PVA (overall, and by type) is described using the observed percentage of subjects with the event and using cumulative incidence curves, estimated where death and weaning from ventilator are competing risks. Assessments of the association of PVA with baseline patient characteristics and ventilator settings using a recurrent events analysis Prentice, Williams, and Peterson gap time (PWP-GT) model^32^, and the correlation of PVA event with hospital mortality and hospital-free days using adjusted logistic regression and multivariable-adjusted linear regression respectively are described in Supplementary Information S3.

### Ethics approval and consent to participate

This study was approved by the Mayo Clinic Institutional Review Board, under the approval number #18-0098365, June 27th, 2019. All research was performed in accordance with relevant guidelines/regulations, and where applicable informed consent was obtained from all participants and/or their legal guardians.

## Results

### Baseline patient characteristics and incidence of PVA throughout the course of mechanical ventilation

A total of 676 patients who underwent 696 episodes of mechanical ventilation were included. Thirteen patients had more than one episode of hospital stay (Supplementary Fig. S1). Demographics, comorbidities, reasons for initial mechanical ventilation, and initial ventilator mode are shown in Table 1. During the entire course of 696 episodes of mechanical ventilation, overall PVA occurred in 170 (24%) episodes (Supplementary Fig. S1). The most common PVA was double triggering 92 (13%) episodes, followed by flow starvation 73 (10%) episodes. The incidence of cycle mismatch 29 (4%) was similar to ineffective effort 27 (4%). Delayed triggering rarely occurred;7 (1%) episodes (Table 1). Double triggering (2.11% vs. 1.09%) and flow starvation (1.63% vs.0.76%) occurred more frequently in volume targeted ventilation (VC) than in pressure targeted ventilation (PC) (both *P* < 0.01) Supplementary Table S1.

**Table 1:** Patient demographics, comorbidities, initial ventilator mode, and number and percentage of all patients experiencing events at any time during mechanical ventilation.

	Overall (N = 696)
Age (year)	64 (52, 72)
**Sex**	
Male	403 (58%)
Female	293 (42%)
Ideal body weight (kg; n = 695)	64.2 (54.3, 73.3)
Apache III score (n = 695)	95 (62, 122)
**Chronic comorbidities**	
History of smoking	395 (57%)
Heart disease	292 (42%)
Lung disease	182 (26%)
Kidney disease	161 (23%)
Immunosuppression	152 (22%)
Neurologic	96 (14%)
Cirrhosis	45 (6%)
**Reason for ventilation (n = 685)**	
Post-surgery	241 (35%)
Pneumonia/sepsis/ARDS	201 (29%)
Neuro	84 (12%)
Other	159 (23%)
**Initial ventilator mode**^a^	
VC	426 (61%)
VC-SIMV	140 (20%)
PC/PC-SIMV	130 (19%)
**Patient ventilator asynchrony (PVA) events**	
Overall PVA^b^	170 (24%)
Double trigger	92 (13%)
Flow mismatch	73 (10%)
Cycle mismatch	29 (4%)
Ineffective effort	27 (4%)
Trigger delay	7 (1%)

Variables are summarized as median (25th percentile, 75th percentile) or number and percentage as appropriate. When all data are not available, numbers with complete data are presented. Data represent 696 episodes of mechanical ventilation in 676 unique patients. In total, 13 patients had greater than 1 episode, with 1 patient having 6 episodes.

^a^Initial ventilator mode reflects the first mode recorded following ventilation start. It may not be the mode at the start time of ventilation.

^b^Overall PVA included flow starvation, double triggering, ineffective effort, delayed triggering and cycle mismatch.

**Figure1:**
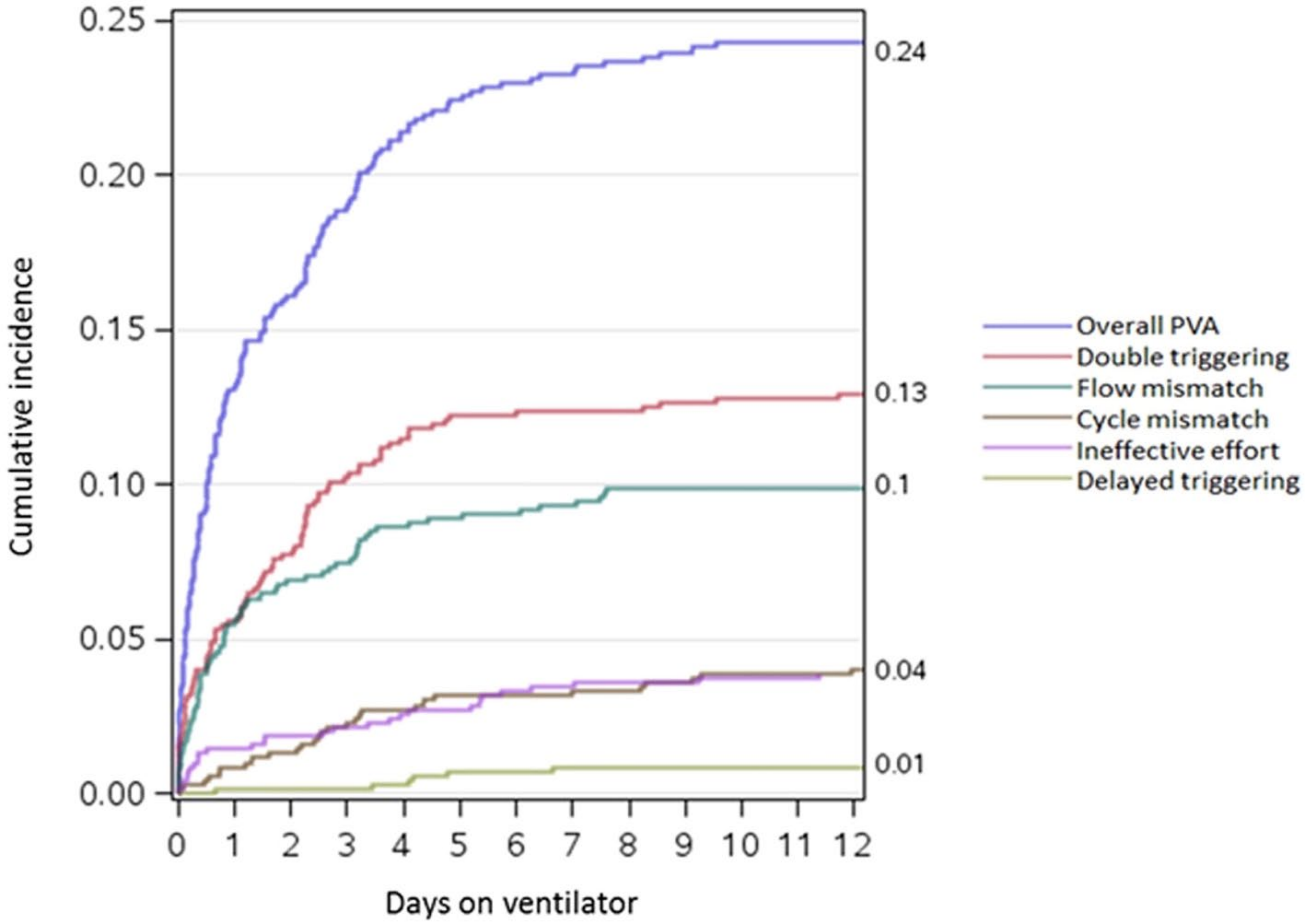
Cumulative incidence of asynchrony over the first 12 days of mechanical ventilation.

The cumulative incidences of patients experiencing all types of PVA at day 12 in Supplementary Table S2, were similar to those during the whole period of mechanical ventilation in Table 1. Figure 1 presents the cumulative incidence of PVA over the first 12 days of mechanical ventilation. The first 5 days were associated with a higher rate of cumulative incidence for double triggering, and the first 8 days with a higher rate for flow starvation. Incidences of cycle mismatch and ineffective effort increased gradually through the first 12 days. Occurrence of delayed triggering started after the third day.

### Association of PVA with baseline patient characteristics and ventilator settings

PWP-GT model was used to analyze the association of time to PVA event with patient characteristics (Table 2) and ventilator settings (Table 3). History of smoking (HR 1.46, 95% CL 1.13–1.89; *P* = 0.004), cirrhosis (HR 1.57, 95% CL 1.06–2.32; *P* = 0.024), and of reasons for initial mechanical ventilation, pneumonia/sepsis/ARDS (HR 1.48, 95% CL 1.01–2.16) and other (HR 1.52, 95% CL 1.02–2.27) compared to post-surgery (*P* = 0.007) were risk factors for overall PVA. However, heart disease (HR 0.65, 95% CL 0.50–0.85; *P* = 0.002) was negatively associated with overall PVA. When analysis for the specific type of PVA, history of smoking, kidney disease, and pneumonia/sepsis/ARDS in comparison with post-surgery were risk factors for double triggering, while heart disease and immunosuppression were associated with decreased risk of this event; cirrhosis and ideal body weight (per 10 kg) were associated with increased risk of flow starvation.

**Table 2:** Association of PVA with baseline patient characteristics using PWP-GT model.

	Overall PVA^a^		Double triggering		Flow mismatch	
	Estimate (95% CI)	P-value	Estimate (95% CI)	P-value	Estimate (95% CI)	P-value
Age (per 10 years)	1.02 (0.94–1.12)	0.575	1.06 (0.94–1.19)	0.338	1.07 (0.95–1.21)	0.279
**Sex**		0.348		0.691		0.840
Female	Referent		Referent		Referent	
Male	1.18 (0.83–1.68)		1.10 (0.69–1.74)		1.06 (0.59–1.90)	
Ideal body weight (per 10 kg)	1.06 (0.92–1.23)	0.407	1.00 (0.82–1.22)	0.984	1.30 (1.05–1.61)	0.018
**Chronic comorbidities**						
History of smoking	1.46 (1.13–1.89)	0.004	1.47 (1.03–2.09)	0.035	1.44 (0.94–2.20)	0.090
Heart disease	0.65 (0.50–0.85)	0.002	0.49 (0.34–0.72)	< 0.001	0.84 (0.53–1.32)	0.442
Lung disease	0.94 (0.74–1.21)	0.634	0.96 (0.65–1.43)	0.841	0.93 (0.57–1.52)	0.764
Kidney disease	1.24 (0.96–1.62)	0.105	1.58 (1.08–2.30)	0.017	0.64 (0.40–1.03)	0.064
Immunosuppression	0.76 (0.52–1.11)	0.157	0.57 (0.36–0.91)	0.019	0.98 (0.59–1.62)	0.933
Neurologic	0.77 (0.51–1.15)	0.194	0.84 (0.48–1.46)	0.536	0.67 (0.35–1.30)	0.235
Cirrhosis	1.57 (1.06–2.32)	0.024	1.38 (0.75–2.54)	0.297	2.14 (1.06–4.31)	0.034
**Reason for ventilation**		0.007		0.026		0.103
Post-surgical	Referent		Referent		Referent	
Pneumonia/sepsis/ARDS	1.48 (1.01–2.16)		1.83 (1.03–3.26)		1.69 (0.99–2.87)	
Neuro	0.67 (0.37–1.20)		0.74 (0.33–1.68)		0.68 (0.26–1.77)	
Other	1.52 (1.02–2.27)		1.62 (0.88–3.00)		1.43 (0.76–2.69)	
**Ventilator mode**		< 0.001		0.003		0.001
VC	Referent		Referent		Referent	
VC-SIMV	0.86 (0.48–1.55)		0.83 (0.39–1.75)		1.13 (0.43–2.99)	
PC	0.50 (0.38–0.68)		0.45 (0.29–0.69)		0.35 (0.21–0.61)	
PC-SIMV	1.47 (0.83–2.60)		2.58 (1.00–6.69)		1.51 (0.42–5.45)	

PWP-GT model : Prentice, Williams, and Peterson gap time. Robust sandwich variance estimates were used to account for the potential within patient correlation. For each model, the terms were assessed for collinearity and functional form.

^a^Overall PVA included flow starvation, double triggering, ineffective effort, delayed triggering and cycle mismatch.

**Table 3:** Association of overall PVA with ventilator settings during different ventilator mode using PWP-GT model.

	Estimate (95% CI)	P-value
**VC ventilator mode**		
VC-Vt per IBW setting (per mL)	1.06 (0.90–1.23)	0.494
Respiratory rate setting	1.02 (0.99–1.05)	0.221
PEEP (cm H_2_O)	1.02 (0.97–1.07)	0.398
**VC-SIMV ventilator mode**		
Peak inspiratory flow setting	1.12 (1.04–1.20)	0.002
Respiratory rate setting	0.93 (0.79–1.10)	0.409
PEEP (cm H_2_O)	0.82 (0.70–0.95)	0.009
**PC ventilator mode**		
Inspiratory time set (per 0.1 s up to 1.2 s)	0.92 (0.82–1.02)	0.100
Inspiratory time set (per 0.1 s after 1.2 s)	1.07 (0.98–1.17)	0.158
Pressure setting (per 1 unit up to 12)	0.95 (0.87–1.04)	0.286
Pressure setting (per 1 unit after 12)	1.10 (1.03–1.17)	0.006
PEEP (per cm H_2_O)	0.98 (0.91–1.05)	0.592

PWP-GT model: Prentice, Williams, and Peterson gap time. As there were different settings in different modes, we did 3 analyses limited to 3 ventilation modes due to rare frequency of PC-SIMV ventilation, including among the 3 analysis a total 666 patients underwent 686 episodes of mechanical ventilation.

Models were fit using data from patients with the given ventilator delivery type (mode) at any point during their episode of mechanical ventilation, with only the time under that delivery mode contributing to the given analysis. All models were adjusted for age, sex, and ideal body weight, history of smoking, heart disease, chronic lung disease, kidney disease, immunosuppression, neurologic disease, cirrhosis, and reason for initiation of mechanical ventilation. In addition to the baseline covariables listed, each model included the mode-specific settings as time-dependent covariables.

Robust sandwich variance estimates were used to account for the potential within patient correlation. For each model, the terms were assessed for collinearity and functional form. When evidence of non-linearity was found, the piecewise linear spline was chosen for ease of interpretation.

Other notes: Each model was stratified by number of prior asynchrony events. Multiple events were allowed and start/stop times were re-started after a change in ventilator mode or an asynchrony event.

Adjusting for baseline characteristics, chronic comorbidities, and reasons for initiation of mechanical ventilation, in patients on synchronized intermittent mandatory ventilation with volume-controlled ventilation (VC-SIMV), increasing peak inspiratory flow setting (HR 1.12, 95% CL 1.04–1.20; *P* = 0.002) was associated with increased risk of overall PVA, while increasing PEEP (HR 0.82, 95% CL 0.70–0.95; *P* = 0.009) was associated with decreased risk of PVA event. In PC ventilation, an inspiratory pressure above PEEP of 12cmH_2_O, was associated with increased risk of PVA event (HR 1.10 per cmH_2_O, 95% CI 1.03–1.17, *P* = 0.006). However, in VC ventilation, no association was observed between ventilator settings and overall PVA event.

### Patient outcome: correlation of PVA with hospital mortality and hospital-free days

Patients with overall PVA, double triggering, and flow starvation were associated with fewer ventilator-free days, longer duration of mechanical ventilation, longer ICU and hospital stay, and higher ICU and hospital mortality than those without (all *P* < 0.01) in Supplementary Table S3.

In the analysis of the effect of PVA on subsequent outcome (length of hospital stay and hospital mortality post-invasive ventilation), 97 patients who died on ventilator were excluded and a total of 599 patients were analyzed. 128 (21.4%) patients experienced at least one PVA event, hospital-free days at day 28 were 15.2 (0.1–21.2) vs. 19.7 (11.8–23.5) days, hospital mortality 14% vs. 7% in patients with and without PVA respectively (Table 4). After adjustment for history of smoking, heart disease, reasons for mechanical ventilation and initial ventilation modes, overall PVA (RR − 4.00; 95% CL − 5.81 to − 2.18; *P* < 0.001), double triggering (RR − 3.12; 95% CL − 5.43 to − 0.81; *P* = 0.008) and flow mismatch (RR − 4.24; 95% CL − 7.01 to − 1.47; *P* = 0.003) were each associated with fewer hospital-free days, but no association was observed between PVA and hospital mortality (*P* > 0.05) in Table 5.

**Table 4:** Post-extubation or -weaning success outcomes (n = 599).

Outcome	Patients without any kind of PVA (N = 471)	Patients with at least one PVA event (N = 128)
28-day hospital-free days, median (Q1, Q3)	19.7 (11.8, 23.5)	15.2 (0.1, 21.2)
Hospital mortality, n (%)	35 (7%)	18 (14%)

Patients who died on ventilator (n = 97) were excluded from the analysis. We describe hospital-free days through 28 days—defined as the number of days alive and out of hospital during 28 days after extubation or weaning success. Weaning success was defined as patients who received tracheostomy and no longer required mechanical ventilation.

*PVA* patient ventilator asynchrony.

**Table 5:** Association between PVA and post-extubation or -weaning success outcomes (n = 599).

	Overall PVA^a^		Double triggering		Flow starvation	
	Estimate (95% CI)	P-value	Estimate (95% CI)	P-value	Estimate (95% CI)	P-value
Hospital-free days^28^	− 4.00 (− 5.81 to − 2.18)	< 0.001	− 3.12 (− 5.43 to − 0.81)	0.008	− 4.24 (− 7.01 to − 1.47)	0.003
Hospital mortality^b^	1.87 (0.98 to 3.58)	0.057	1.01 (0.39 to 2.59)	0.989	1.37 (0.54 to 3.48)	0.511

Models are linear regression for hospital-free days and logistic regression for hospital mortality. Models account for correlation between multiple observations per subject with robust variance estimates using the generalized estimating equations approach). All models are adjusted for history of smoking, heart disease, reason for intubation, and initial ventilation delivery mode. Patients who died on ventilator (n = 97) were excluded from the analysis.

^a^Overall PVA included flow starvation, double triggering, ineffective effort, delayed triggering and cycle mismatch.

^b^Estimates are odds ratios and represent the increased odds of hospital mortality associated with the given asynchrony type.

## Discussion

The current study examines the incidence and consequences of PVA in medical, surgical, and medical-surgical patients managed across a number of general and subspecialty ICUs staffed by providers of varying background (internal medicine/Pulm CC, anesthesia, surgery). The main findings include: (1) Overall, PVA was common. Double triggering was most prevalent, followed by flow starvation; (2) Risk factors for the development of PVA—and double triggering specifically—include a history of smoking, sepsis, pneumonia, or ARDS as etiology of respiratory failure. PC ventilation was associated with a lower overall incidence of PVA, double triggering, and flow starvation compared to VC; (3) Double triggering, flow starvation, and the total number of PVA per patient were associated with worse outcome and fewer hospital-free days.

In our study, the overall prevalence of PVA in adult, critically ill patients over their entire course of mechanical ventilation was 24%. The most prevalent PVA was double triggering, followed by flow starvation. This finding is, to some extent, consistent with previous studies reporting that double triggering occurs in most mechanically ventilated patients^11,13,15,33^. However, others have shown that the incidence of PVA varies widely with the most common being ineffective effort^1,2,4,5,7^. This may be explained by differences in study population (e.g., COPD, trauma, medical or surgical patients), observation time (e.g., 1–10 min, 30 min, or one day), detection method (e.g., clinical assessment, waveform continuously monitored, detection of esophageal pressure and electrical activity of the diaphragm), and ventilator settings^1,2,4,5,7^.

PVA can occur throughout the course of mechanical ventilation and varies widely over time^15,33^. In a recent proof of concept study, Marchuk et al.^34^ developed a Hidden Markov model to predict the time course of PVA and inferred the probability that the number of PVA events would be above a given threshold, based on discrete time-series data in 51 mechanically ventilated patients. Here we report the cumulative incidence of PVA, identifying the first 12 days after mechanical ventilation initiation as a critical period over which the risk for development of any PVA event increases. The first 5 days appear to be a critical time with a high likelihood of developing double triggering; Over the first 8 days, flow starvation. This finding may suggest that critically ill, mechanically ventilated patients could benefit from closer monitoring of those with a higher risk of PVA over this time period, to enable early identification and intervention upon PVA to improve patient–ventilator interaction.

### Factors associated with overall PVA

Patient factors may predict PVA. A history of smoking, cirrhosis, and pneumonia/sepsis/ARDS as etiology of respiratory failure, as opposed to a post-surgery status, were positively associated with overall PVA events. Conversely, heart disease was negatively associated with overall PVA. Several studies reported that COPD, ARDS, and greater severity of illness favor the occurrence of PVA^3,11,33^. In PC ventilation, higher inspiratory pressure (> 12 cmH_2_0 above PEEP), and in VC-SIMV mode, higher inspiratory flow were associated with a higher risk of PVA, while higher PEEP levels were associated with lower risk. During VC ventilation, no association was observed between ventilator settings and overall PVA event. Robinson et al.^24^ found ventilator asynchrony was more common in SIMV with set breathing frequencies of > 10 breaths/min in trauma patients. Similar to the previous studies^30,35,36^, the use of PC ventilation was associated with better patient–ventilator interaction than VC ventilation, but requires careful monitoring to avoid delivery of larger than targeted volumes.

### Factors associated with double triggering and flow starvation

Double triggering occurs when there is a mismatch between set tidal volume or inspiratory time and patient’s ventilatory demand^16,29,31,37^. Risk factors include a history of smoking, chronic kidney disease, and pneumonia/sepsis/ARDS, while chronic heart disease and immunosuppression had a reduced risk of double triggering. We speculate that kidney disease may cause acidosis, resulting in increased central respiratory drive. Pulmonary function impairment might be more severe in patients with pneumonia/sepsis/ARDS than those patients intubated in the postoperative period, and this may lead to high ventilatory demand.

Flow starvation occurs when ventilator flow rate is less than patient demand. Our results demonstrate a positive correlation between cirrhosis and ideal body weight with flow starvation. We speculate that patients with greater ideal body weight may need higher flow and that cirrhosis might cause increased ventilatory demand or neural drive through liver-lung cross talk.

We confirm previous reports^12,30,35,36^ that VC ventilation is associated with more frequent double triggering and flow mismatch events, perhaps due to inadequate tidal volume or flow as a result of strict limitation by operators. However, de Haro et al. found a higher percentage of double cycling occurred in PCV than in VCV with constant flow or decelerated flow^33^. A plausible explanation for this discrepancy could be related to differences in study population and ventilator settings. We did not analyze the influence of the ventilator mode-specific settings on the occurrence of double triggering and flow mismatch due to the limited number of events.

### Outcome

Patients in our cohort who developed PVA were associated with fewer ventilator-free days, longer ICU and hospital stay, and higher ICU and hospital mortality than those without. However, after patients who died on ventilator were excluded, with the analysis of the effect of PVA on subsequent outcome (length of hospital stay and hospital mortality post-invasive ventilation), overall PVA, double triggering and flow mismatch each independently predicted fewer hospital-free days, but no association was observed between PVA and hospital mortality after adjustment for history of smoking, heart disease, reasons for mechanical ventilation and initial ventilation modes.

Nevertheless, examining PVA in trauma patients^24^ or in the early phase of weaning^38^, with a short observation showed that asynchrony index (number of PVA events/total respiratory rate × 100) > 10% was not associated with adverse clinical outcome; Rue et al. using Bayesian analysis found that patient ventilator asynchrony was positively associated with alive discharge^39^. What’s more, Thille and Blanch, et al.^12,15^ showed that asynchrony index > 10% was associated with prolonged duration of mechanical ventilation, or higher ICU and hospital mortality. Additionally, de Wit and Vaporidi et al.^17,18^ found that ineffective triggering index ≥ 10% or clusters of ineffective triggering were correlated with a worse outcome. Given the differences in population, timing and duration of observation, and the definition of asynchrony employed, the varying outcomes and the erratic results^40^, the relationships between asynchronies and outcomes still need to be further validated.

Our study examined the relationships of double triggering, flow starvation, and patient outcome. Both were associated with worse outcomes. As expected, the total delivered volume during double triggering events was much larger than the set/targeted tidal volume, often double or more a normal breath^30,33^, which could lead to overinflation. Stronger spontaneous inspiratory effort during flow starvation can cause harmful transpulmonary pressure swings, which might lead to occult pendelluft and consequent regional lung overdistension^41,42^. Those mechanisms might cause ventilator induced lung injury and worsen outcomes^42^. Our study indicated the association of PVA with a poorer prognosis, but whether the relationship between PVA and outcome is causative or associative requires further investigation.

### Strengths

To our knowledge, this is the first and largest study to systemically investigate the incidence of overall and specific types of PVA, their risks, and associated outcomes among a heterogeneous population of ICU patients in a large, academic institution, where well trained RTs routinely manage PVA per local standardized clinical practice guidelines. This study used a PWP-GT approach to estimate the correlation of time to PVA events with factors related to patient characteristics, ventilator settings—time-dependent covariates throughout the entire course of mechanical ventilation; and analyzed the prediction of time to PVA events on the subsequent outcomes after extubation or weaning success.

### Limitations

Our study has several limitations. First, this is a single-center, retrospective cohort study, which may limit generalizability. Second, PVA detection relied on ventilator waveform analysis by RT’s at designated timepoints, thus the incidence of PVA is likely underestimated. Third, reverse triggering, the activation of diaphragm muscles by a ventilator initiated breath, may produce several cycling desynchronies including double triggering or double cycling, which causes breath stacking. As reverse triggering was not evaluated as a specific PVA, its contribution to the incidence of double triggering was not evaluated. Though software that provides continuous monitoring and automatic detection of PVA may be available in the near future, it is not currently part of routine clinical practice. This study analyzed a real-world method for identifying PVA applicable to any bedside intensive care practice. Lastly, for reasons of statistical power, factors associated with double triggering and flow starvation related to ventilator settings were not analyzed.

## Conclusion

PVA are common, with double triggering and flow starvation encountered most often among critically-ill patients undergoing at least 12 h of invasive mechanical ventilation. Occurrence is associated with worse outcome, including fewer hospital free days. Patient characteristics including day of mechanical ventilation and etiology of respiratory failure may predict development of PVA and help to identify at-risk individuals for closer monitoring and early intervention. Further investigations are needed to determine whether the relationship between PVA and outcome is associative or causal.

## Supplementary Information

Supplementary Information.

## Abbreviations

PVA: Patient–ventilator asynchrony
PEEP: Positive end of expiratory pressure
ARDS: Acute respiratory distress syndrome
COPD: Chronic obstructive pulmonary disease
PWP-GT: Prentice, Williams, and Peterson gap time
EMR: Electronic medical record
VC: Volume targeted ventilation
PC: Pressure targeted ventilation
VC-SIMV: Synchronized intermittent mandatory ventilation with volume-controlled ventilation
PC-SIMV: Synchronized intermittent mandatory ventilation with pressure controlled ventilation
