# Supplementary material for: Etiology, incidence, and outcomes of patient–ventilator asynchrony in critically-ill patients undergoing invasive mechanical ventilation
Source: Sci Rep. 2021 Jun 11;11:12390. doi: 10.1038/s41598-021-90013-z (PMC8196026; doi:10.1038/s41598-021-90013-z)
Supplement: Supplementary file 1 — Supplementary Information. [file 41598_2021_90013_MOESM1_ESM.pdf]

Etiology, Incidence, and Outcome of Patient-Ventilator Asynchrony in Critically-Ill Patients Undergoing Invasive Mechanical Ventilation

Zhou, Yongfang M.M.[2], Steven R. Holets R.R.T., L.R.T.[3], Man Li M.D.[4], Gustavo A. Cortes-Puentes M.D.[1], Todd J. Meyer R.R.T., L.R.T. [3], Andrew C. Hanson B.S.[5], Phillip J. Schulte, Ph.D [5], Richard A. Oeckler M.D., Ph.D.[1*]

Author affiliations

1. Division of Pulmonary and Critical Care Medicine, Mayo Clinic Rochester, MN, United States; 2. Department of Critical Care Medicine, West China Hospital, Chengdu, China; 3.Department of Respiratory Therapy, Mayo Clinic Rochester, MN, United States; 4. Department of Information Technology, Mayo Clinic Rochester, MN, United States; 5. Department of Health Science Research, Mayo Clinic, Rochester, MN, United States.

[*]**Corresponding author:** Richard A. Oeckler, MD, PhD

Address: Mayo Clinic

Division of Pulmonary & Critical Care Medicine

200 1st St SW

Rochester, MN 55902 USA

Email: Oeckler.Richard@mayo.edu.

Telephone: +1 (507) 284-2158

Additional file method and results

Contents

Additional file S1.Ventilator Asynchrony Recognition Quiz……………………………………….Section.1

Additional file S2. The classification of ventilation modes…………………………………………Section.2

Additional file S3. Statistical Analysis………………………………………………………………………. Section.3

Additional file S4. Results……………………………………………………………………………………… Section.4

Figure S1……………………………………………………………………………………4.1

Table S1……………………………………………………………………………………4.2

Table S2……………………………………………………………………………………4.3

Table S3……………………………………………………………………………………4.4

S1.Ventilator Asynchrony Recognition Quiz

Identify the type of asynchrony in the following waveforms

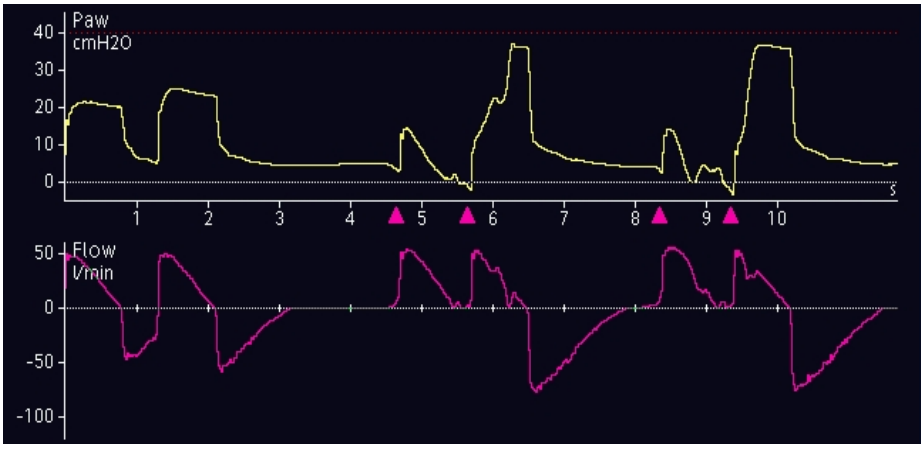

1. The above waveform represents:
    a. delayed triggering
    b. ineffective efforts
    c. double triggering
    d. flow starvation
2. If the above represents a stable post op cardiac surgery patient with a fast track weaning order the most appropriate intervention would be to:
    a. Re-sedate
    b. Change flow profile
    c. Place on spontaneous mode (pressure support, ASV)
    d. Increase tidal volume

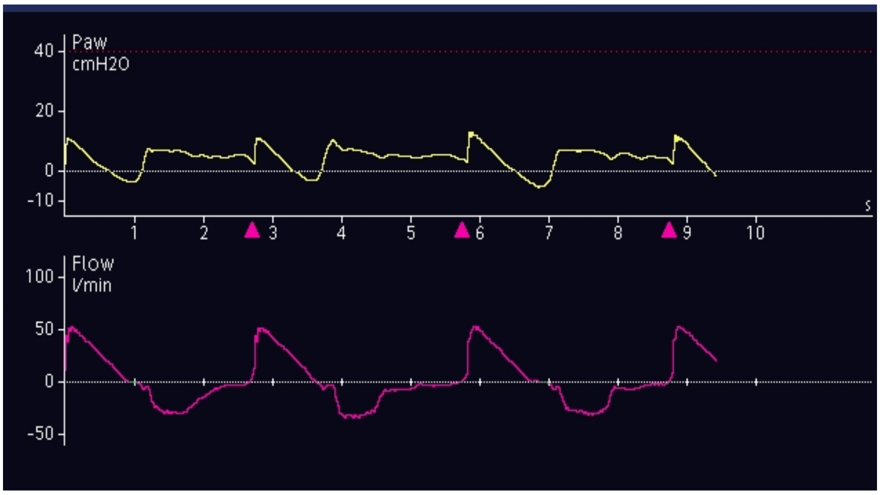

3. The above waveform represents:
    a. Cycle mismatch (premature or prolonged cycling)
    b. Flow mismatch (flow starvation)
    c. Trigger delay
    d. Double triggering
4. The patient appears uncomfortable. What intervention will improve patient comfort without changing mode
    a. Increase rate
    b. Increase PEEP
    c. Change flow or flow profile
    d. Decrease trigger sensitivity

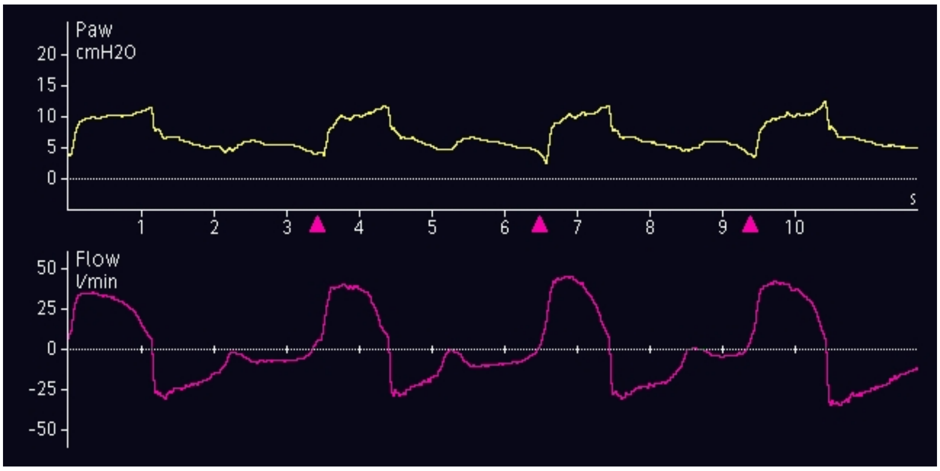

5. The above waveform represents:

a. Ineffective efforts (wasted efforts)
b. Cycling mismatch
c. Flow mismatch
d. Double triggering

6. The above patient has severe COPD. To improve synchrony the most appropriate intervention would be to:

a. Decrease flow
b. Increase trigger sensitivity
c. Increase PEEP
d. Increase inspiratory pressure

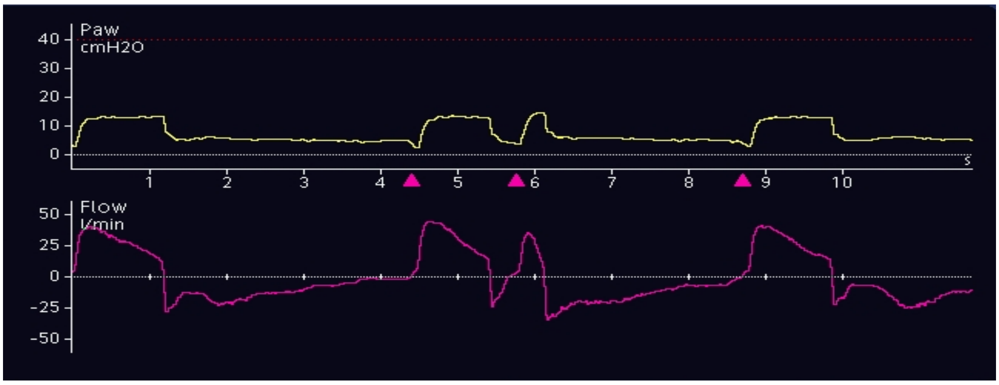

7. The above waveform represents:

a. Cycling mismatch (premature)
b. Flow starvation
c. Cycling mismatch (delayed)
d. Auto cycling

8. To improve synchrony and increase tidal volume the most appropriate intervention would be to:

a. Increase flow
b. Decrease expiratory trigger sensitivity (↓%)
c. Decrease inspiratory trigger sensitivity
d. Increase expiratory trigger sensitivity (↑%)

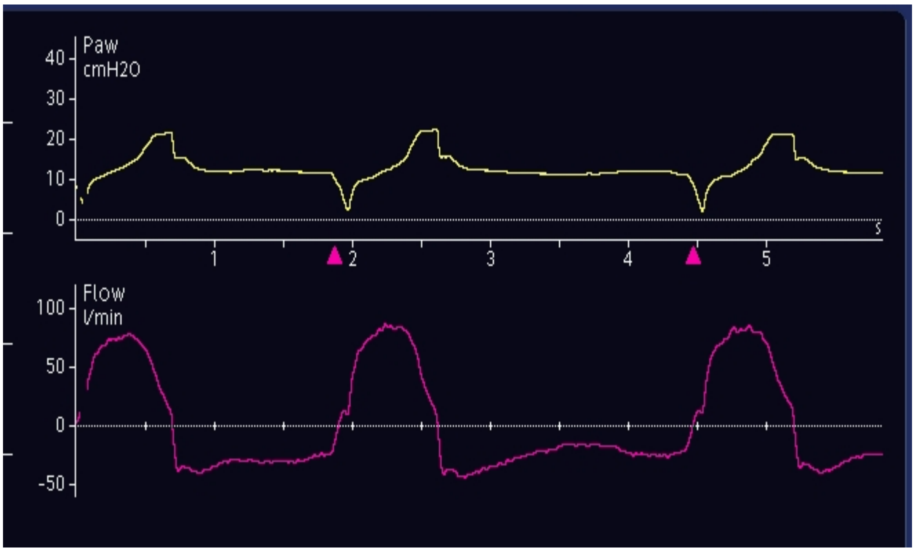

9. The above waveform represents a patient on PS 10, PEEP 10cmH_20, trigger sensitivity of -5 cmH_20. The above is an example of what type of asynchrony?
   a. Ineffective effort (wasted effort)
   b. Flow mismatch (to high)
   c. Trigger delay
   d. Cycle mismatch (prolonged)
10. To decrease the patient's work of breathing the most appropriate intervention would be to:
    a. Increase set flow rate and increase pressure trigger sensitivity
    b. Change to flow trigger and increase rise time
    c. Change to flow trigger and decrease rise time
    d. Decrease pressure trigger sensitivity and increase rise time

S2. The classification of ventilation modes

Ventilator modes included: VC: Volume targeted ventilation (A/C-VCV: Assist/control-volume control ventilation, or CMV: Continuous mandatory ventilation); VC-SIMV: Synchronized intermittent mandatory ventilation with volume controlled ventilation; PC: Pressure targeted ventilation (Pressure controlled ventilation, pressure supported ventilation, biphasic positive airway pressure; and adaptive support ventilation); PC-SIMV: Synchronized intermittent mandatory ventilation with pressure controlled ventilation.

S3. Statistical Analysis

Incidence of PVA (overall, and by type) is described using the observed percentage of subjects with the event and using cumulative incidence curves, estimated where death and weaning from ventilator are competing risks.

In the first aim, we assess the association between patient characteristics, medical history, and initial ventilator mode on the incidence of overall PVA. As patients may have multiple, repeated PVA events, we used a recurrent events analysis Prentice, Williams, and Peterson gap time (PWP-GT) model (32). The PWP model uses a robust covariance to obtain standard errors accounting for recurrent events on subjects. Covariates in this model were specified by investigators and included age, sex, ideal body weight, smoking history, history of heart disease, lung disease, kidney disease, immunosuppression, neurologic conditions, cirrhosis, the reason for ventilation, and initial ventilator mode. Covariates were assessed for collinearity using variance inflation factors and functional form of continuous variables by comparing to a more flexible restricted cubic spline relationship. No violations were identified for collinearity or functional form. Similarly, PWP-GT models were fitted to specific endpoints for each of double triggering and flow starvation. Results are reported as hazard ratios (HR), with 95% confidence intervals (CI) and p-values, for the association between each characteristic and the intensity/hazard for the PVA event.

In the second aim, we sought to assess the relationship between ventilator settings and overall PVA, separately for each ventilator mode. Ventilator settings are changed during the course of ventilation, sometimes in response to PVA events. For patients on VC ventilation, we use PWP-GT models, adjusted for baseline covariates listed previously, to assess the relationship between ventilator settings-tidal volume per ideal body weight (Vt per IBW), respiratory rate, and PEEP and subsequent PVA events. Changes in ventilator settings were updated over time as time-dependent variables. Analyses were also performed for VC-SIMV ventilation for the settings-inspiratory flow, respiratory rate, and PEEP, and for PC ventilation for the settings-inspiratory time and pressure, and PEEP. A Functional form of continuous settings was assessed in these models as described previously. When non-linear relationships were detected, we identified piecewise linear splines that fit the data well.

Finally, we describe post-invasive ventilation outcomes. We describe hospital-free days through 28 days – defined as the number of days alive and out of hospital during 28 days after extubation or weaning success. Weaning success was defined as patients who received tracheostomy and no longer required

mechanical ventilation. Patients who died on ventilator are excluded, while patients who died in hospital post-invasive ventilation are assigned the worst outcome. The association between PVA event and post-invasive ventilation hospital mortality was assessed using adjusted logistic regression. Hospital-free days were compared between those with and without PVA event using multivariable-adjusted linear regression. Analyses were also performed to assess the association by individual type of PVA event for double triggering and flow starvation. All analyses used a robust sandwich covariance approach to account for correlated observations for subjects with multiple distinct ventilator periods meeting inclusion criteria for the study.

Analyses were performed in SAS statistical software v9.4. No sample size calculation was performed *a priori*.

S4. Results

Figure S1. Flow chart represents the incidence of different type of PVA and distribution of ventilatory modes.

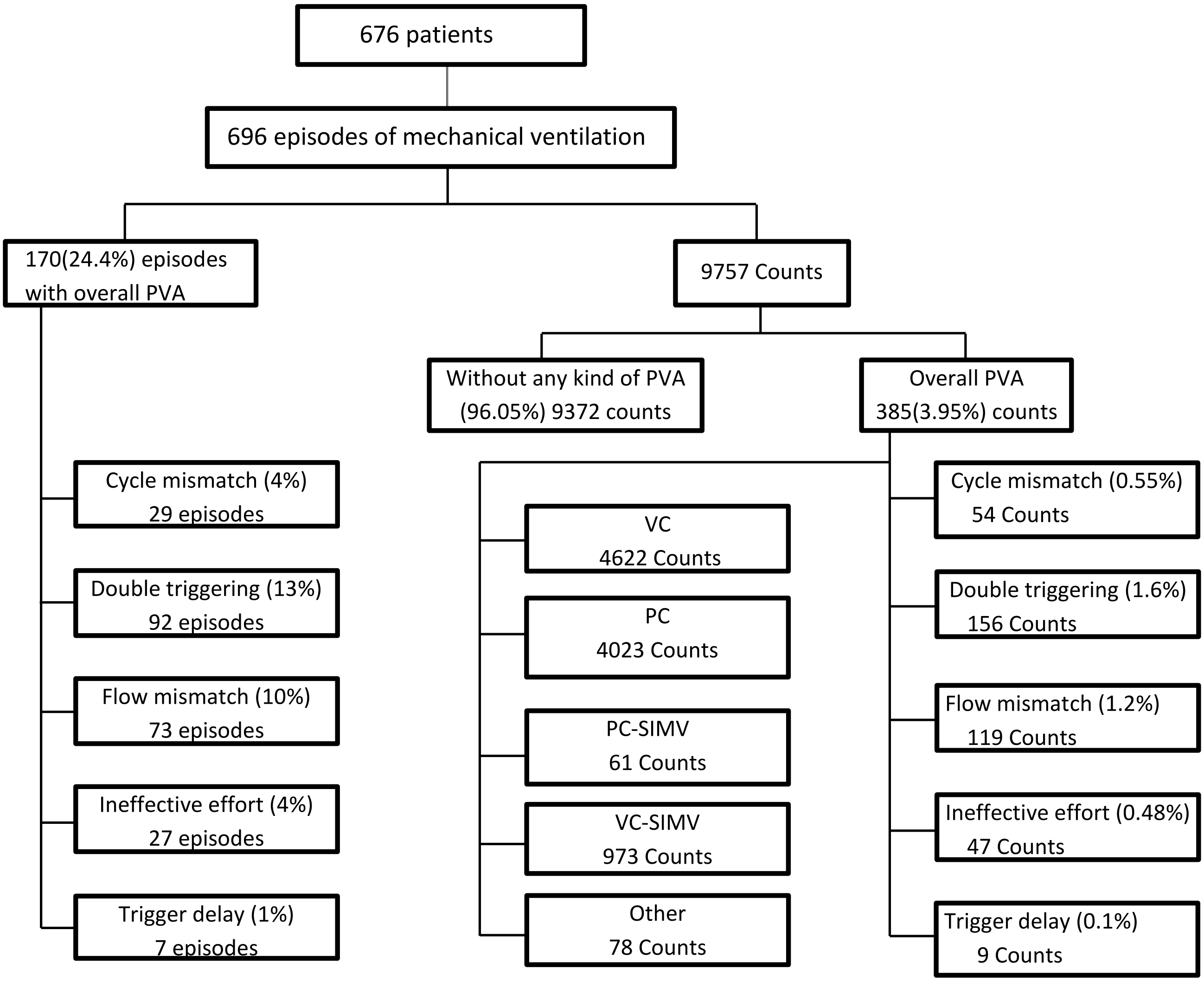

PVA: patient ventilator asynchrony, PC: pressure targeted ventilation, VC: volume targeted ventilation, PC-SIMV: synchronized intermittent mandatory ventilation with pressure controlled ventilation, VC-SIMV: synchronized intermittent mandatory ventilation with volume controlled ventilation. Other: There was no any kind of specific mode marked in 78 counts, which did not have any kind of PVA event.

Table S1. The incidence of PVA in different mode

	VC	PC	VC-SIMV	PC-SIMV
Overall PVA	4.65%(215/4622)	3.26%(131/4023)*	2.67%(26/973)	21.31%(13/61)
Double trigger	2.11%(95/4622)	1.09%(43/4023) *	1.04%(10/973)	(8/61)
Flow starvation	1.63%(73/4622)	0.76%(30/4023) *	1.25%(12/973)	(4/61)

PC was compared with VC, *P<0.05.

Table S2. Cumulative incidence estimates at 12 days*

Endpoint	*Estimate (95% CI)*
**Overall PVA**	0.243 (0.212 to 0.275)
Double trigger	0.129 (0.106 to 0.155)
Flow mismatch	0.099 (0.078 to 0.123)
Cycle mismatch	0.040 (0.027 to 0.057)
Ineffective effort	0.039 (0.026 to 0.055)
Trigger delay	0.009 (0.004 to 0.018)

* Estimates and corresponding confidence limits are for the cumulative incidence of patients experiencing the given event during the first 12 days of mechanical ventilation.

Table S3. Outcomes

Variable	Patients with any kind of PVA (n=170)	Patients without PVA (n=526)	*P* value	Patients with double triggering (n=92)	Patients without double triggering (n=604)	*P* value	Patients with flow starvation (n=73)	Patients without flow starvation (n=623)	*P* value
Duration of mechanical ventilation, d, median (IQR)	3.8(1.8-7.8)	1.6(0.8-3.2)	<0.001	4.0(2.1-8.8)	1.7(0.9-3.7)	<0.001	4.4(1.8-9.4)	1.8(0.9-3.8)	<0.001
Vent free days -28-day, d, median (IQR)	22.0(0-25.9)	26.2(22.8-27.1)	<0.001	20.7(0-25.1)	26.1(21.6-27.1)	<0.001	20.3(0-25.4)	26.0(21.2-27.1)	<0.001
ICU Length of stay, d, median (IQR)	7.1(3.2-14.4)	3.9(2.2-7.7)	<0.001	7.3(4.1-15.0)	4.1(2.2-8.3)	<0.001	7.7(3.2-15.5)	4.2(2.3-8.5)	<0.001
ICU mortality, n (%)	50(29.4)	72(13.7)	<0.001	31(33.7)	91(15.1)	<0.001	24(32.9)	98(15.7)	<0.001
Hospital Length of stay, d, median (IQR)	15.7(7.6-32.1)	11.2(6.2-20.7)	<0.001	15.6(7.7-32.7)	11.4(6.3-21.4)	0.017	18.5(8.6-35.2)	11.5(6.3-21.3)	0.001
Hospital mortality, n (%)	*60(35.3)*	*90(17.1)*	*<0.001*	*33(35.9)*	*117(19.4)*	*0.003*	*29(39.7)*	*121(19.4)*	*<0.001*
